# Behavioral Therapies for Treating Female Sexual Dysfunctions: A State-of-the-Art Review

**DOI:** 10.3390/jcm11102794

**Published:** 2022-05-16

**Authors:** Gemma Mestre-Bach, Gretchen R. Blycker, Marc N. Potenza

**Affiliations:** 1 Facultad de Ciencias de la Salud, Universidad Internacional de La Rioja, 26006 Logroño, La Rioja, Spain; gemma.mestre@unir.net; 2 College of Nursing, University of Rhode Island, Kingston, RI 02881, USA; gblycker@uri.edu; 3 Department of Psychiatry, Yale University School of Medicine, New Haven, CT 06510, USA; 4 Child Study Center, Yale University School of Medicine, New Haven, CT 06510, USA; 5 Connecticut Mental Health Center, New Haven, CT 06519, USA; 6 Connecticut Council on Problem Gambling, Wethersfield, CT 06109, USA; 7 Department of Neuroscience, Yale University, New Haven, CT 06510, USA

**Keywords:** female sexual dysfunction, cognitive behavioral therapy, mindfulness, treatment, female orgasmic disorder, female sexual interest/arousal disorder, genito-pelvic pain/penetration disorder

## Abstract

Many possible factors impact sexual wellbeing for women across the lifespan, and holistic approaches are being utilized to promote health and to address sexual concerns. Female sexual dysfunction disorders, including female orgasmic disorder, female sexual interest/arousal disorder and genito-pelvic pain/penetration disorder, negatively impact quality of life for many women. To reduce distress and improve sexual functioning, numerous behavioral therapies have been tested to date. Here, we present a state-of-the-art review of behavioral therapies for female sexual dysfunction disorders, focusing on empirically validated approaches. Multiple psychotherapies have varying degrees of support, with cognitive-behavioral and mindfulness-based therapies arguably having the most empirical support. Nonetheless, several limitations exist of the studies conducted to date, including the frequent grouping together of multiple types of sexual dysfunctions in randomized clinical trials. Thus, additional research is needed to advance treatment development for female sexual dysfunctions and to promote female sexual health.

## 1. Introduction

Sexual health is an important part of sexual wellbeing for women [1]. The World Health Organization (WHO) defines sexual health as “a state of physical, emotional, mental and social well-being in relation to sexuality,” with “the possibility of having pleasurable and safe sexual experiences, free of coercion, discrimination, and violence [2].” There are a growing number of holistic frameworks for promoting sexual wellbeing [3,4]. A model that expands on this holistic and biopsychosocial definition to provide a comprehensive public health approach to promote sexual wellbeing includes four foundational pillars which are the domains of sexual health, sexual pleasure, sexual wellbeing, and sexual justice [1]. The pillar of sexual health includes fertility management, sexual violence prevention, prevention and management of sexually transmitted infections, and sexual function, desire, and arousal. The pillar of sexual wellbeing includes sexual safety and security, sexual respect, sexual self-esteem, resilience in relation to sexual experience, forgiveness of past sexual experience, comfort with sexuality, and self-determination in one’s sexual life [1].

Multiple variables may influence sexual wellbeing for women throughout the lifespan. Sexual concerns are natural and common, and therefore having appropriate care and support to meet these needs is important. To address this necessity for women, the International Society for the Study of Women’s Sexual Health developed a process of care (POC) that includes recommendations for clinicians to provide universal screenings to assess for sexual concerns. A suggested approach includes a four-step model of opening dialog, “eliciting the story, naming/reframing attention to the problem, empathic witnessing of the patient’s distress and the problem’s impact, and referral or assessment and treatment” [5].

The International Classification of Diseases 11th Revision (ICD-11) chapter on Conditions Related to Sexual Health includes classifications that reflect an integrated understanding of the mind/body connection while acknowledging the value of pleasure to be included in the promotion of holistic treatment and approaches for sexual wellbeing [6]. According to the fifth edition of The Diagnostic and Statistical Manual of Mental Disorders (DSM-5) [7], the classifications of female sexual dysfunctions require that an individual encounters clinically significant disturbance in the physiological ability to respond sexually or experience pleasure while experiencing conducive conditions that are also providing adequate and effective stimulation. Since sexual responsivity is influenced by a confluence of intrapersonal, interpersonal, and sociocultural factors, clinical assessment should be multidimensional in order to discern favorable conditions for each individual. If conducive conditions are not actualized, then treatment addressing the problem or difficulties may be indicated, though the criteria for a sexual dysfunction diagnosis may not be met.

Specific diagnoses for female sexual dysfunction that appear in the DSM-5 include female orgasmic disorder, female sexual interest/arousal disorder, and genito-pelvic pain/penetration disorder (Table 1), although some conditions (e.g., substance/medication-induced sexual dysfunction) may be relevant to both males and females. Diagnostic criteria to be considered and assessed may relate to etiology and differential diagnosis, and may influence the treatment protocol and plan. The assessment process may also involve identifying subtypes, including specifiers that may provide information about the onset and etiology of the sexual difficulty. Categorizing the problem as lifelong indicates the problem has been present since the beginning of sexual experiences. An acquired designation describes a problem that has developed after having experienced healthy sexual functioning. Understanding whether the problem is experienced in a generalized way sexually is useful to rule out dependent variables that may influence sexual functioning and satisfaction, including, for example, quality of stimulation, solo experiences, partner-specific outcomes, and relational factors. The situational subtype is important to differentiate when, with whom, and under what circumstances the sexual difficulty occurs [7].

Studies show that most women report orgasm during masturbation [8,9], which typically includes varieties of clitoral stimulation. Therefore, in assessing situational factors involving experiencing orgasm during vaginal-penile intercourse for females, it is important to phrase questions that ask about concurrent clitoral stimulation. For example, a study showed that women reported frequency of orgasm with intercourse that included assisted clitoral stimulation 51–60% of the time compared to orgasm 21–30% of the time without concurrent clitoral stimulation. Concurrent clitoral stimulation and body movement are associated with higher likelihood of orgasm during vaginal penetration [10]. A cross-sectional national probability survey of 3017 U.S. women surveyed types of movement techniques in which women engage to make penile or sex toy vaginal penetration more pleasurable that include “angling” for 87.5% of women, “rocking” for 76% of women, “shallowing” for 84% of women, and “pairing” manual clitoral stimulation with penetration for 67% of women [11].

Historically, multiple sources have created barriers to knowledge about female sexual functioning and pleasure [12]. More accurate information about the anatomy and physiology of the female clitoral complex, which is homologous to the glans and corpus cavernosa of the male penis [13] and just as central to experiencing of sexual pleasure [14], was only reported in 1998 [15]. Other barriers to knowledge involve sociocultural contexts, with gendered sexual scripts that may contribute to beliefs and behaviors that prioritize male sexual entitlement to pleasure [16] when operating from conceptualizations about sex that center around a phallocentric focus on vaginal-penile intercourse for women who have sex with men (WSM) [17]. A study examining 30 various traits and behaviors that were correlated with orgasm frequency during sexual experiences found that heterosexual women were least likely (65%) to say they almost always experienced orgasm when sexually intimate during the last month, followed by bisexual women (66%), and lesbian women were the most likely (86%) among women to experience orgasm. Heterosexual men were the most likely to report orgasm (95%), followed by gay men (89%) and bisexual men (88%) [18]. 

These findings highlight the evidence of gendered sociocultural factors contributing to an orgasm gap discrepancy between men and women, and that heterosexual women may have the potential to experience more pleasure and higher rates of orgasm if the factors underlying the disparity are addressed [18]. This underscores the importance, in the assessment and evaluation of female sexual difficulties, not to pathologize an appropriate physiological response (i.e., lack of sexual arousal or absence of orgasm during experiences of insufficient sexual stimulation, solo or partnered) and to consider the multiplicity of factors contributing to sexual functioning concerns.

In this article, we present a state-of-the-art review of behavioral therapies that have empirical support in the treatment of female sexual dysfunction disorders. We focus on two types of psychotherapies, cognitive-behavioral and mindfulness-based therapies, given the studies conducted to date on these approaches. We consider the existing data with respect to the support for these treatments and highlight existing gaps where further research appears indicated.

## 2. Methods

We conducted a state-of-the-art review. As such, we aimed to conduct a comprehensive review of existing studies and describe the findings in a narrative format. In this manner, we sought to provide a current understanding of behavioral therapies with empirical support for the treatment of female sexual dysfunction disorders. Additionally, we sought to identify target areas for future investigation.

## 3. Results: Data Supporting Approaches for Treating Female Sexual Dysfunctions

### 3.1. Cognitive Behavioral Therapy

Cognitive-behavioral therapy (CBT) is a short-term, symptom-focused psychotherapy that considers that thoughts and decisional actions are malleable treatment targets. A central objective is to break maladaptive cognitive-affective-behavioral chains [19]. Some main CBT strategies may include cognitive restructuring (modification of maladaptive thoughts associated with emotional distress), behavioral activation (increasing involvement with activities), exposure (having gradual contact with factors that generate anxiety), and problem solving [20]. When addressing female sexual dysfunctions, CBT has typically included other non-pharmacological strategies, such as directed masturbation, sex therapy, sensate focus, communication training, systematic desensitization, anxiety-reduction strategies, bibliotherapy, counseling, and psychoeducation, among others [21]. Some aspects that are addressed in CBT through these tools are [22]: (a) frequency of sexual behavior, (b) sexual fantasies, (c) beliefs, (d) environmental and interpersonal factors, and (e) symptoms of anxiety and depression (see Table 2).

CBT appears effective for treating female sexual dysfunctions, especially if sexual dysfunction is considered as frequency of sexual stimulation, sexual responses, and sexual contingencies [23]. CBT may help women with sexual dysfunctions to identify which factors enhance and which factors generate sexual limitations, as well as to restructure maladaptive thoughts about their sexuality, and reduce the tendency to avoid certain sexual behaviors [24]. CBT has been proposed for different types of female sexual dysfunctions [25]. Although the central objective of CBT in these dysfunctions is an improvement in sexual function and sexual satisfaction, there are specific aspects to be addressed in each form of female sexual dysfunction. For example, in the case of female orgasmic disorder, the central goal of CBT is for women to improve their abilities to experience orgasm, increase their sexual satisfaction, and reduce high levels of anxiety often associated with this dysfunction. CBT focuses on promoting changes in women’s attitudes and behaviors through exercises and techniques, such as Kegel exercises, communication skills training, sex education, directed masturbation, and systematic desensitization. It has been suggested that CBT is effective in improving orgasmic response [26,27,28,29,30].

In the case of female sexual interest/arousal disorder, CBT aims to increase rewards in order to increase motivation for sexual activities. Three controlled trials support CBT for addressing the symptomatology of this disorder [31,32]. In addition, CBT has been found to be efficacious improving factors associated with the disorder, such as marital functioning [33].

Regarding genito-pelvic pain/penetration disorder, CBT aims to achieve pain control in sexual contexts [34]. CBT may help increase the frequency of sexual intercourse, decrease the fear of coitus, and enhance successful non-coital penetration, in comparison with no treatment [35]. In addition, CBT for genito-pelvic pain/penetration disorder appears effective in reducing anxious symptomatology and improving marital harmony [36].

In addition to evaluating the efficacy of CBT in each particular female sexual dysfunction, some studies have evaluated CBT in all of them together. For example, McCabe [37] administered a 22-week CBT program to 54 women with female sexual dysfunction and observed that CBT was associated with improved levels of sexual dysfunction. For example, the percentage of participants with anorgasmia was reduced from 67% (n = 36) at baseline to 11% after the intervention. Moreover, participants reported greater perceptions and positive attitudes toward sex after treatment, as well as less self-perceived sexual failure and less interference of sexual dysfunction in their relationship. Stephenson et al. [38] conducted a randomized clinical trial involving 99 women aged 18–65 years who met DSM-IV-TR criteria for hypoactive sexual desire disorder, female sexual arousal disorder, and/or female orgasmic disorder. Participants were randomly assigned to one of the four treatment groups: (1) CBT; (2) Ginkgo biloba extract (an ancient tree extract that seems to facilitate blood flow, to relax smooth muscle tissue, and to influence nitric oxide systems); (3) CBT + Ginkgo biloba extract; and (4) placebo. The manualized CBT consisted of eight weekly individual sessions with the aim of focusing on the awareness of the physiological sensations present during sexual arousal, as well as reducing the negative anticipation of sexual experiences. In order to achieve these objectives, techniques such as cognitive restructuring, exposure, or progressive muscle relaxation were used. No differences were observed between the CBT and CBT + Ginkgo biloba extract groups in terms of treatment outcome. Relationship satisfaction at baseline was a relevant factor, as it was associated with greater improvements in both sexual satisfaction and distress levels, although it did not predict changes in participants’ sexual functioning. Thus, although those women who reported relationship dissatisfaction may show improvements in sexual functioning, these may not be associated with greater sexual satisfaction and subjective well-being.

The efficacy of CBT has been contrasted with the efficacies of other treatments, such as supportive psychotherapy (SPT), cognitive-behavioral bibliotherapy, pharmacotherapy, and waiting lists. In the case of SPT, although it appears that both approaches are effective in reducing the pain severity of vulvodynia, CBT appears to be more effective than SPT in reducing pain severity during physical examination, and in improving sexual function in women with vulvodynia [39]. Regarding cognitive-behavioral bibliotherapy and waiting lists, these approaches appear less efficacious than CBT in the case of lifelong vaginismus. More successful intercourse was observed with CBT after treatment and at 12 months follow-up, compared to the other two options, in a sample of 117 women [40]. CBT may also be more effective than sildenafil in improving female sexual function in women with arousal and orgasm dysfunction. Comparing 43 women who received CBT with 43 women who received sildenafil (50 mg), Omidi et al. [41] observed that those who received CBT reported improvements in multiple aspects of sexual functioning (except arousal, orgasm, and lubrication), as well as in marital satisfaction.

CBT may be helpful for women presenting with more than one sexual dysfunction and may be considered within sexual health frameworks. A sexual health model has been proposed that includes the following components [30]: (a) “Talking about sex”, to enhance the ability to talk about one’s sexual values, behaviors, and history in order to negotiate a mutually satisfying sexual relationship; (b) “culture, gender, and sexual identity”, to recognize and question the impact that female gender and culture has on their own sexual identities; (c) “sexual anatomy and functioning”, to understand and accept female sexual functioning; (d) “sexual health care and safer sex”, to learn about the body and recognize changes associated with health; (e) “challenges and barriers to sexual health”, to identify possible barriers to sexual health, such as psychiatric illness or sexual abuse; (f) “body image”, to work on body self-acceptance in order to enhance comfort with sexuality; (g) “masturbation and fantasy”, to enhance self-knowledge of the body and discover which aspects facilitate arousal and orgasm; (h) “positive sexuality”, to learn about female-sexuality-oriented models that value gender differences in arousal and orgasm; (i) “intimacy and relationships”, to improve relationship problems prior to sexual difficulties; and (j) “spirituality and religion”, to find a congruence between sexual behaviors and values and religious or spiritual beliefs.

### 3.2. Mindfulness-Based Approaches

Mindfulness has been understood as an “awareness that emerges through paying attention, on purpose, in the present moment, and nonjudgmentally, to the unfolding of experience moment by moment” [42]. Mindfulness-based approaches have begun to be used in clinical practice, either as an adjunct to other therapies (usually CBT) or as a stand-alone therapy. An approach with arguably the most evidence to date is mindfulness-based cognitive therapy (MBCT) [43]. MBCT is a treatment program that includes education related to cognitive modeling, training in decentering, and promotion of abilities or tendencies to perceive cognitions and feelings as mental events, not necessarily as reality. In addition, mindfulness may promote acceptance, compassion, and better management of ruminative and intrusive thoughts.

Although MBCT was initially designed to address other pathologies, some authors suggested that the integration of mindfulness training in female sexual dysfunctions could be effective for the improvement of well-being and sexual function [44,45,46]. Therefore, MBCT for sexuality (MBCT-S) has been tested, for example, in women with sexual interest/arousal disorder. Paterson et al. [47], in their uncontrolled pilot trial, tested MBCT-S in 26 women with sexual interest/arousal disorder. MBCT-S, as a modification of more traditional mindfulness practices, includes sex therapy exercises performed in a mindful way, with an aim of training equanimity for interoceptive sensations during sexual practices. The 8 weekly group sessions included aspects such as body scan; stretch and breath; breath, body, sounds, and thoughts; mindfulness of breath; working with difficulty; and working with sensations in the body. Participants showed significant improvements in sexual desire, sex-related distress, and sexual functioning. Likewise, mindfulness and depressive symptomatology also improved significantly after treatment and mediated the improvement in sexual functioning. In their randomized trial, Brotto et al. [48] used the MBCT-S proposed by Paterson et al. [47], and compared the efficacy of group MBCT + sex education with that of group supportive sex education and therapy in a sample of 148 women with sexual interest/arousal disorder. Similar significant improvements were observed in both groups when comparing sexual desire and arousal at baseline and post-treatment assessments. Likewise, patients in the MBCT-S group reported greater decreases in sexual distress and greater improvements in relationship satisfaction and ruminations about sex, compared to the other group. Similarly, the efficacy of MBCT-S was observed by Halvaiepour et al. [49] in 45 women with sexual interest/arousal disorder. In the case of MBCT for provoked vestibulodynia, the intervention has also shown promising results, similar to those obtained by CBT [50].

The use of brief MBCT interventions has been suggested, with promising results for women with sexual distress, sexual difficulties, and histories of childhood sexual abuse [51], gynecologic cancers and sexual dysfunctions [52,53], low sexual desire and arousal [54], sexual difficulties and multiple sclerosis and spinal cord injuries [55], and sexual dysfunctions after risk-reducing salpingo-oophorectomy [56]. Similarly, a brief intervention has also been proposed for women with sexual desire and arousal difficulties, involving three 90-min sessions every 2 weeks. The sessions include psychoeducation on the multifactoriality of female sexual disorders and their prevalence; introduction to mindfulness-based exercises and in-session mindfulness-based practices; cognitive challenging of maladaptive sexual thoughts; and incorporation of mindfulness into sexual exercises, among other aspects. Several studies have reported possible improvements in all domains of participants’ sexual responses, as well as reductions in sexual distress [57,58]. In addition, studies based on this brief intervention that include psychoeducation have reported a significant increase in genital subjective sexual arousal concordance following the intervention [59]. However, more evidence is needed.

Other studies have recently tested the efficacy of online or video-based mindfulness-based approaches. For example, Hucker and McCabe [60], in their randomized study, tested the efficacy of the Pursuing Pleasure program (an online CBT intervention that includes mindfulness training and online chat groups). This program was administered to 26 women experiencing sexual difficulties related to desire, arousal, orgasm, and/or pain. A wait-list control group (n = 31) was also included. The treatment group showed significant improvements in their sexual difficulties, except for sexual pain, as well as significant decreases in distress and frequencies of sexual problems, which were maintained at follow-up. Similar results were reported in another study [61]. Brotto et al. [62] tested the efficacy of an online program consisting of 12 sessions that included psychoeducation and mindfulness-based elements in 46 women with sexual difficulties resulting from gynecologic cancers. The authors reported improvements in sexual functioning, sex-related distress and mood after treatment, which were maintained at follow-up.

Another randomized study tested the efficacy of video-based, self-administered MBCT compared with CBT in 65 women who reported difficulties reaching orgasm [63]. MBCT included a total of seven videos, which were received by the participants once a week and could be viewed as many times as they wished. Each video began with a mindfulness exercise and then gave instructions on how to be mindful during sexual activities (e.g., mindful of one’s genitals or pelvic movements). Each of the videos ended with the prescription of individual and/or couple-based exercises related to sexuality for the following week. Patients had the option of contacting a researcher as many times as they needed throughout the intervention. After the interventions, participants in both groups reported significant improvements in sexual functioning compared to baseline (16% increase in the CBT group and 9% in the MBCT group). The effect was maintained at the second month of follow-up. Significant reductions in sexual distress and improvements in orgasm, desire, arousal, and sexual satisfaction were also observed in both groups. However, reductions in sexual pain only occurred in the group receiving CBT. The efficacy of an online mindfulness-based intervention, compared to online CBT interventions, and a waitlist control group, is also being tested in a randomized controlled trial involving 266 women with hypoactive sexual desire dysfunction [64]. The mindfulness-based approach (MIND) includes formal and informal exercises that have been adapted for sexual dysfunctions, organized in eight modules: (a) “Introduction to mindfulness”; (b) “being present in the moment: formal and informal mindfulness”; (c) “bodily sensations, sitting meditation”; (d) “mindfulness towards thoughts”; (e) “being present during sexual activity”; (f) “letting go”; (g) “detached awareness”; and (h) “addressing difficulties”. The modules aim for participants to consolidate their daily mindfulness practice, to accept negative thoughts and emotions and to acquire detachment from experiences related to the body, cognitions, and emotions. However, the results will not be available until later in or after 2022.

Other mindfulness-based approaches have been proposed in recent years, such as the “Integrated Mindfulness for Provoked Vestibulodynia” (IMPROVED) treatment. This is a mindfulness-based approach in a group format of four sessions every two weeks that includes, among other aspects, CBT, psychoeducation and mindfulness meditation skills with the aim of managing the genital pain of women with vestibulodynia. Although this brief program seems to be promising for the reduction of pain catastrophizing, hypervigilance, and sex-related distress, as well as for increasing pain self-efficacy [65], more evidence is needed.

### 3.3. Other Approaches

Other treatments for female sexual dysfunctions have been suggested, such as acceptance and commitment therapy [66] and psychoeducational interventions [52,67]. In addition, the effects of traditional Chinese approaches to treating female sexual dysfunctions (besides mindfulness) have also been begun to be studied [68,69], mainly including acupuncture [70,71,72,73,74,75], yoga [75,76,77,78,79], and herbal products [80]. Although these eastern techniques may have preliminary promising results in the treatment of female sexual dysfunctions, the literature is scarce, so it is not possible to draw solid conclusions about their efficacies [75].

## 4. Discussion

This state-of-the-art/narrative review summarizes studies and findings relating to the psychotherapeutic approaches used in the treatment of female sexual dysfunction disorders. As described above, both CBT and mindfulness-based approaches have empirical support in the treatment of female sexual dysfunction disorders.

CBT appears to be an effective approach for all female sexual dysfunction disorders. In general, it is intended to improve both sexual function and satisfaction. CBT allows women to identify factors associated with dysfunctions, as well as to restructure maladaptive cognitions regarding sexuality. CBT also allows women to expose themselves to sexual behaviors that they had tended to avoid. However, for each sexual dysfunction disorder, specific aspects may be important to address, such as sex education, masturbation, and systematic desensitization.

In contrast to the efficacy of CBT with other therapies for female sexual dysfunction disorders, it has been suggested that the efficacy of CBT is greater than supportive psychotherapy, cognitive-behavioral bibliotherapy, sildenafil, and wait-list conditions. However, it should be noted that more clinical trials are needed to specifically test the effectiveness of each of the techniques used for each female sexual dysfunction disorder. This process should involve investigation and identification of the active components related to the mechanisms of action of CBT for specific female sexual dysfunction disorders. In this process, individual differences of treatment participants should also be considered within a precision-medicine framework.

Similar considerations hold for MBCT. MBCT could be an effective approach for female sexual dysfunction disorders since it has been associated with an improvement in sexual function and well-being of women. In addition, it has been suggested that MBCT could reduce sex-related distress and that these improvements may be maintained in the follow-up stages of treatment. MBCT may operate through different mechanisms than CBT (for example, the cognitive mechanisms employed in CBT may differ from those employed in MBCT, with respect to utilizing alternate coping mechanisms versus feeling and allowing, for example). As such, MBCT may operate differently from CBT, and some individuals may have preferences for one approach or the other, or may find the skills gleaned from one approach versus the other easier to learn and implement. Additionally, the two approaches may offer complementary strategies that may enhance the likelihood for bettering treatment outcomes. Of note, both approaches may provide individuals seeking treatment with skills that they may learn and use over the longer term.

In addition to CBT and MBCT, other interventions for female sexual dysfunctions have been proposed, such as psychoeducational interventions, acceptance and commitment therapy, acupuncture, yoga and herbal products. However, studies on the efficacy of these approaches are scarce, so no solid conclusions may be drawn in this regard at present.

However, many studies to date have limitations, especially with respect to grouping together of individuals with different disorders that may respond differently to specific interventions. As such, more research is needed that employs more homogeneous samples and studies these groups over time in sufficient sample sizes to make more definitive statements about which treatments may work best for women with specific sexual dysfunction disorders. Further, such investigations should include measures that provide insight into the mechanisms of action of the therapies, with a focus on the proposed active ingredients of the approaches.

## 5. Limitations and Future Studies

The studies included in this review have several limitations. First, numerous studies addressing female sexual dysfunctions do not specify details of the treatment protocols, simply whether it was CBT, mindfulness-based, or another form of treatment, so comparisons of findings should be made with caution. Second, controlled studies for each of the female sexual dysfunctions are lacking. Third, some studies include women with different sexual dysfunctions, without taking into account that each female sexual dysfunction may have unique clinical features. Finally, the narrative nature of the present review permits coverage of heterogeneous sources and methodologies used in different studies. However, the included studies may have biases associated with the state-of-the-art/narrative approach used in this review. We believe that our intention to comprehensively review the topic of behavioral treatments for female sexual dysfunction disorders justifies the approach of a state-of-the-art review. However, future studies in this area could address the potential biases and limitations of this type of design and conduct a systematic review using the PRISMA guidelines.

## 6. Conclusions

This state-of-the-art review considers behavioral therapies tested in the treatment of female sexual dysfunction disorders. Less research into and consequent understanding of female sexual functioning has been conducted and achieved, respectively, as compared with male sexual functioning [81,82,83]. More research is needed into sexual dysfunctions, especially for females. Although CBT and mindfulness-based therapies appear promising for women experiencing sexual dysfunctions, data for specific disorders are limited. Identifying safe, beneficial, efficacious interventions with limited potential for adverse effects for women with female sexual dysfunction disorders holds promise for improving quality of life for many women. 

## Figures and Tables

**Table 1:** Diagnostic and Statistical Manual of Mental Disorders (DSM-5) Female Sexual Dysfunction Disorders.

DSM-5 Female Sexual Dysfunction Disorders	Diagnosing Specific Female Sexual Dysfunction Disorders *	Other Conditions to Consider when Making Diagnoses of Specific Female Sexual Dysfunction Disorders
Female Orgasmic Disorder; 302.73 (F52.31)	Significant delay, infrequency, absence, or reduced intensity of orgasms in all/most sexual experiences with clinically significant distress over 6 months or more. Important additional considerations: - Barriers to orgasm are not due to lack of clitoral stimulation during vaginal penetration, a mental disorder, a medication/substance, history of abuse or interpersonal or sociocultural factors. - Consider whether an orgasm was experienced under any situation previously. - Diagnosis is based on subjective, self-reports from women.	1. Nonsexual mental disorders. 2. Substance/medication use. 3. Other medical condition. 4. Interpersonal factors. 5. Other sexual dysfunctions.
Female Sexual Interest/Arousal Disorder; 302.72 (F52.22)	Absent or markedly reduced sexual arousal or interest for at least 6 months with clinically significant distress as reflected by: 1. Lacking or low interest in sexual activity with reduced or no sexual or erotic thoughts. 2. Diminished openness to creating a sexual experience and/or being receptive to a partner’s sexual initiation. 3. Diminished or absent sexual arousal or pleasure during most or all sexual experiences. 4. Diminished or absent sexual responsivity to adequate intrapersonal, interpersonal, or external sexual cues. Additionally, sexual dysfunction is not better attributed to a mental disorder, relational distress, other life stressors, a medication/substance, history of abuse or interpersonal or sociocultural factors. Important additional considerations: - Desire discrepancy with a partner is not sufficient for diagnosis, although assessing for interpersonal contexts contributing to experience and symptoms is relevant to identifying etiology of distress or concerns. - With asexual self-identification, no diagnosis is made.	1. Nonsexual mental disorders. 2. Substance/medication use. 3. Other medical condition. 4. Interpersonal factors. 5. Other sexual dysfunctions. 6. Inadequate or absent sexual stimulation.
Genito-Pelvic Pain/Penetration Disorder; 302.76 (F52.6)	Experiencing difficulties with one or more of the following for at least 6 months with clinically significant distress: 1. Challenges to vaginal penetration during sexual activity. 2. Significant pain with attempted vaginal penetration. 3. Significant fear or anxiety about experiencing pain in anticipation of vaginal penetration, during or after vulvovaginal touch or attempted penetration. 4. Significant reflexive or involuntary muscular contraction of the pelvic floor muscles during attempted vaginal penetration.	1. Other medical condition (pelvic inflammatory disease, endometriosis, etc.) 2. Somatic symptom and related disorder. 3. Inadequate sexual stimulation.

* Specify: Lifelong or Acquired, Generalized or Situational, Mild/Moderate/Severe; for additional information, see the Fifth Edition of the Diagnostic and Statistical Manual.

**Table 2:** Female sexual dysfunctions and CBT.

Female Sexual Dysfunction	CBT Aims	Possible Components of the CBT	CBT Effects
Female Orgasmic Disorder	- To promote changes in attitudes and thoughts - To reduce anxiety - To increase orgasmic ability and sexual satisfaction	- Sex education - Cognitive restructuring - Systematic desensitization - Sensate focus - Communication training - Kegel exercises - Directed masturbation	- Higher likelihood to experience orgasm by masturbation and/or with coitus
Female Sexual Interest/Arousal Disorder	- To increase rewarding experiences to promote motivations for engaging in sexual activity - To approach other aspects of female sexual functioning, such as arousal response and lubrication, ability to experience orgasm, or reduction of pain - To improve skills of erotic stimulation - To improve couples’ relationships	- Sex education - Cognitive restructuring - Tools from sensate focus therapy or sex therapy - Communication exercises - Emotional communication skills training - Sexual fantasy training - Orgasm consistency training	- Decreased symptoms of the disorder - Improvements in cognitive, behavioral, and marital functioning - Greater sexual satisfaction
Genito-Pelvic Pain/Penetration Disorder	- To focus on pain and sexuality - To achieve pain control in sexual contexts - To reduce catastrophic fear of pain - To (re)establish satisfying sexual functioning - To reduce muscle contraction in the pelvic floor - To promote lubrication during sexual intercourse	- Sex education - Progressive muscle relaxation - Abdominal breathing - Kegel exercises - Vaginal dilatation - Distraction techniques including focusing on sexual imagery - Rehearsal of coping self-statements - Communication skills training - Cognitive restructuring - Systematic desensitization	- Reduced pain during intercourse - Improved sexual functioning - Reduced fear of coitus and avoidance behavior - Reduced negative penetration beliefs

CBT: Cognitive behavioral therapy.
